# Supplementary figures and images for: Protection promotes energetically efficient structures in marine communities
Source: PLoS Comput Biol. 2023 Dec 21;19(12):e1011742. doi: 10.1371/journal.pcbi.1011742 (PMC10769090; doi:10.1371/journal.pcbi.1011742)

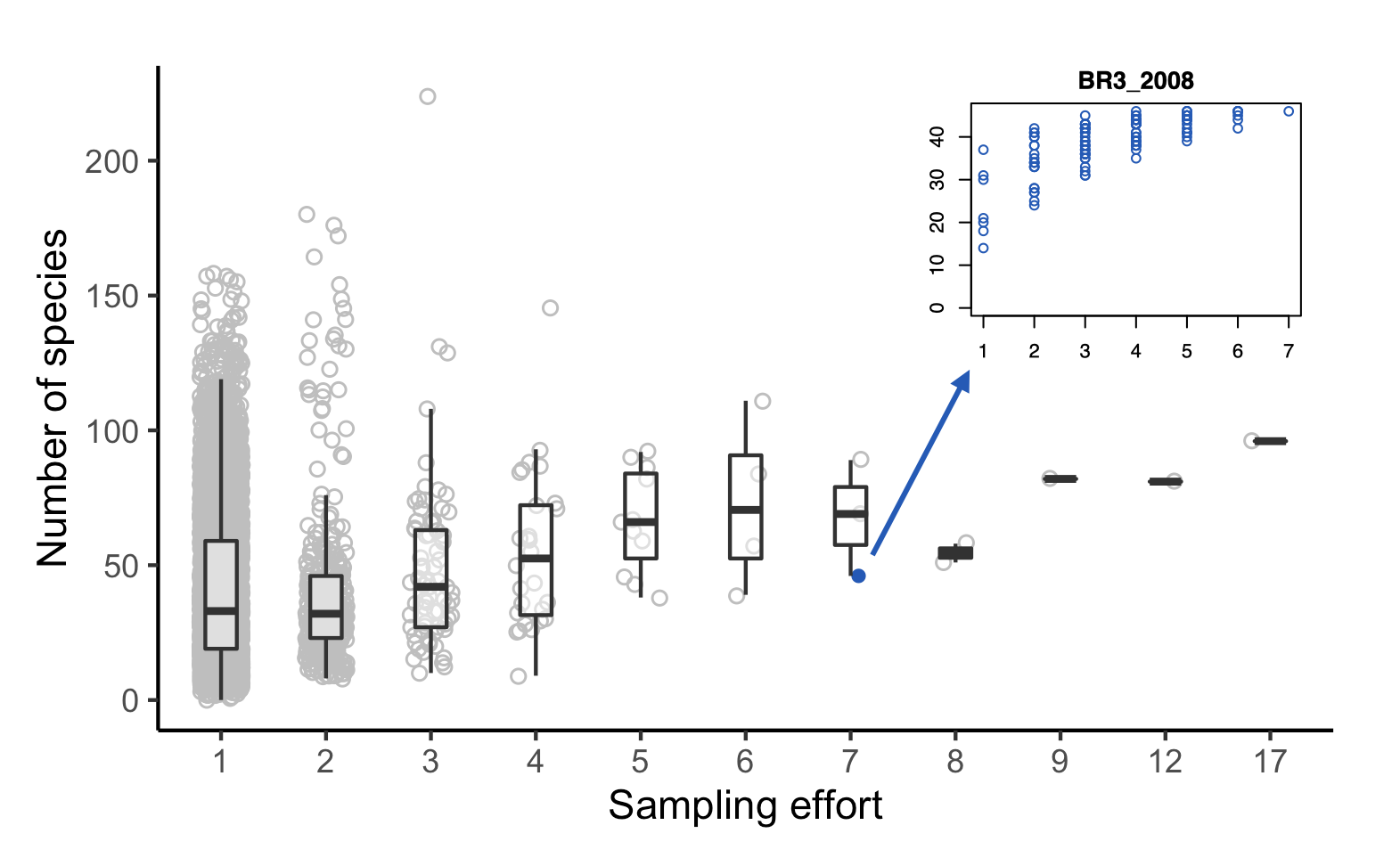

Supplement: S1 Fig — The grey circles represent communities aggregated in a given location across a year. The majority of communities were sampled once per year, and only a small fraction of the aggregated communities were sampled more than once a year (sampling effort >1). For communities sampled more than once in a given year, we conducted a rarefaction analysis to estimate the effect of sampling effort on species richness by resampling communities and then plotting the number of species in each constructed community against sampling effort (an example shown in the top right panel). (TIFF) [file pcbi.1011742.s001.tiff]

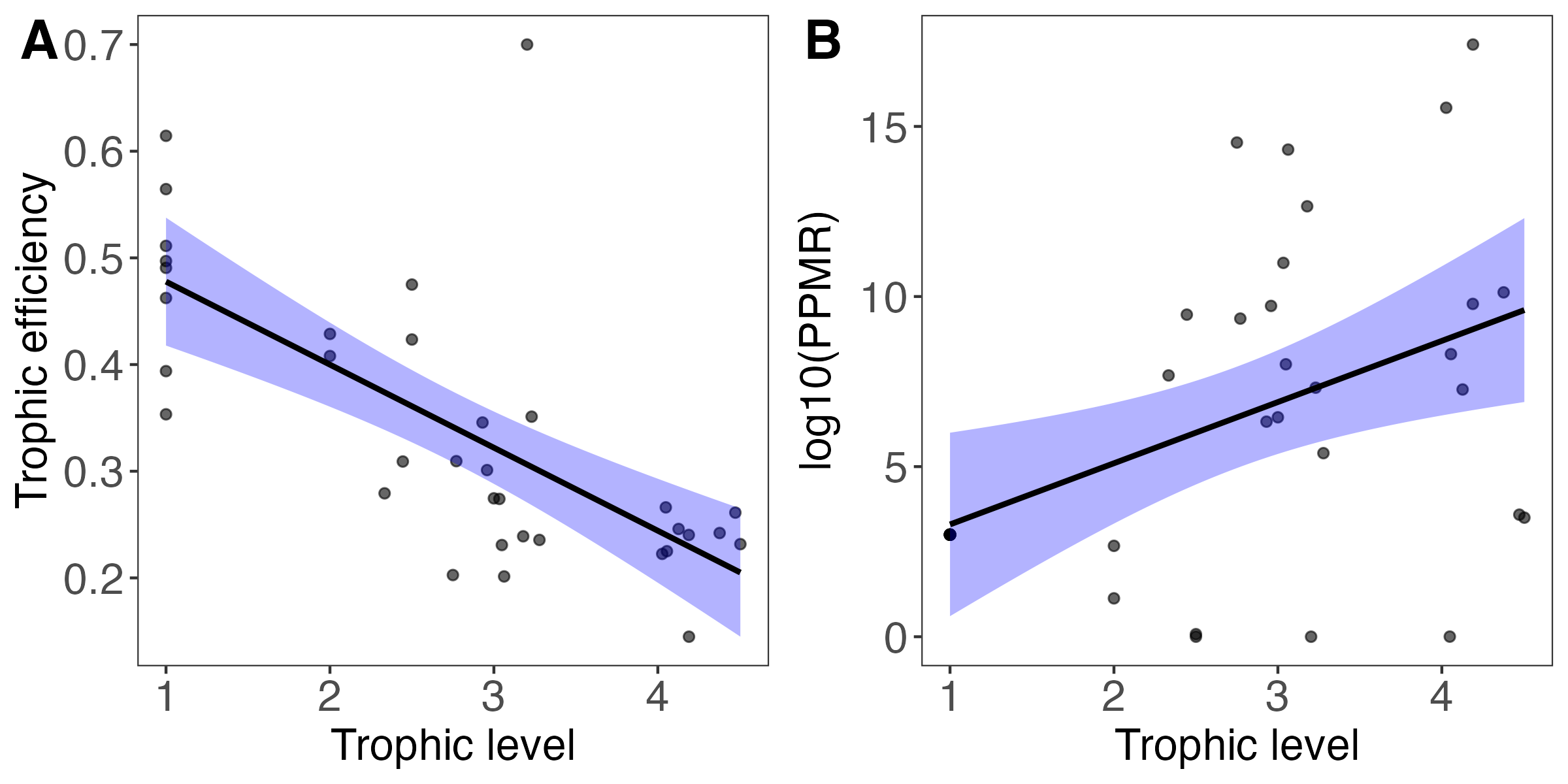

Supplement: S2 Fig — Panel (A) shows an example of trophic efficiencies in a simulated community (TEmax = 0.7). On average, trophic efficiency decreases with trophic level. Panel (B) shows that the average predator-prey mass ratio (PPMR) increases with trophic level. (TIFF) [file pcbi.1011742.s002.tiff]

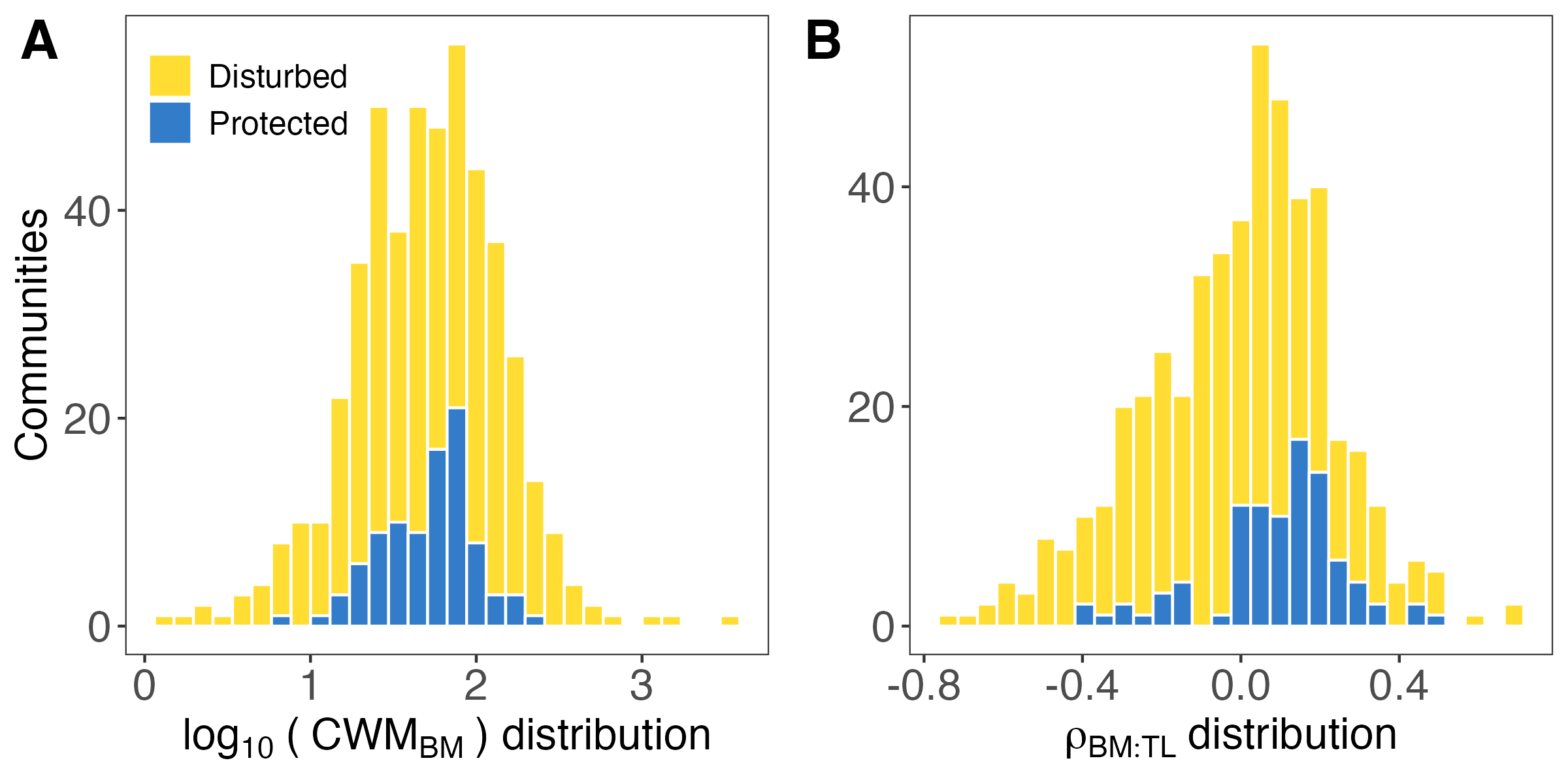

Supplement: S3 Fig — (A) Species body sizes were weighted by species abundance for each community. The average community-weighted mean of body masses (CWMBM) in protected and disturbed areas show similar values. (B) The distribution of Spearman’s rank correlation coefficient between average species body mass and trophic levels for empirical communities shows that empirical body masses do not always increase with trophic level. The average species trophic level was obtained from Fishbase database [63]. (TIFF) [file pcbi.1011742.s003.tiff]
