# Supplementary material for: Protection promotes energetically efficient structures in marine communities
Source: PLoS Comput Biol. 2023 Dec 21;19(12):e1011742. doi: 10.1371/journal.pcbi.1011742 (PMC10769090; doi:10.1371/journal.pcbi.1011742)
Supplement: S1 Table — We used the Kolmogorov–Smirnov test to compare the empirical distributions of species richness across communities with different levels of sampling effort (SE). Two samples are considered not drawn from the same distribution if the p-values <α, where we set α = 0.05. (DOCX) [file pcbi.1011742.s004.docx]

|  | **Distribution 1** | **Distribution 2** | **KS test (p-value)** |
| --- | --- | --- | --- |
| 1 | Species richness of communities with SE > 1 | Species richness of communities with SE = 1 | 7.688 × 10^−4^ |
| 2 | Species richness of all communities | Species richness of communities  with SE = 1 | 0.995 |
| 3 | Species richness of all communities | Species richness of communities  with SE > 1 | 2.594 × 10^−3^ |
| 4 | Species richness of all communities | Species richness of communities  with SE > 2 | 9.809 × 10^−6^ |
| 5 | Species richness of communities  with SE > 1 | Species richness of communities  with SE > 2 | 1.343 × 10^−4^ |
| 6 | Species richness of communities  with SE > 2 | Species richness of communities  with SE > 3 | 0.222 |
| 7 | Species richness of communities  with SE > 3 | Species richness of communities  with SE > 4 | 0.698 |

Table S1: **Comparison of empirical richness distributions across sampling efforts.** We used the Kolmogorov–Smirnov test to compare the empirical distributions of species richness across communities with different levels of sampling effort (SE). Two samples are considered not drawn from the same distribution if the p-values < 0.05.
