# Supplementary material for: Protection promotes energetically efficient structures in marine communities
Source: PLoS Comput Biol. 2023 Dec 21;19(12):e1011742. doi: 10.1371/journal.pcbi.1011742 (PMC10769090; doi:10.1371/journal.pcbi.1011742)
Supplement: S2 Table — We use G2 test of conditional independence [53], where we reject independence when the p-value <0.05. (DOCX) [file pcbi.1011742.s005.docx]

|  | **Variable X** | **Variable Y** | **Conditions S_XY_** | **G^2^ test (p-value)** |
| --- | --- | --- | --- | --- |
| 1 | Human density | Structure | - | 0.052 |
| 1a | Human density | Structure | MPA | 0.807 |
| 1b | Human density | Structure | Coral | 0.076 |
| 1c | Human density | Structure | TSA | 2.290 × 10^−4^ |
| 1d | Human density | Structure | MPA, Coral | 0.715 |
| 1e | Human density | Structure | MPA, TSA | 0.396 |
| 1f | Human density | Structure | Coral, TSA | 1.498 × 10^−4^ |
| 1g | Human density | Structure | MPA, Coral, TSA | 0.435 |
| 2 | Human density | MPA | - | 6.515 × 10^−5^ |
| 2a | Human density | MPA | Structure | 1.834 × 10^−3^ |
| 2b | Human density | MPA | Coral | 2.021 × 10^−5^ |
| 2c | Human density | MPA | TSA | 5.291 × 10^−9^ |
| 2d | Human density | MPA | Structure, Coral | 9.540 × 10^−4^ |
| 2e | Human density | MPA | Structure, TSA | 4.124 × 10^−5^ |
| 2f | Human density | MPA | Coral, TSA | 4.221 × 10^−10^ |
| 2g | Human density | MPA | Structure, Coral, TSA | 2.646 × 10^−5^ |
| 3 | Human density | Coral | - | 0.174 |
| 3a | Human density | Coral | Structure | 0.198 |
| 3b | Human density | Coral | MPA | 0.023 |
| 3c | Human density | Coral | TSA | 0.115 |
| 3d | Human density | Coral | Structure, MPA | 0.056 |
| 3e | Human density | Coral | Structure, TSA | 0.037 |
| 3f | Human density | Coral | MPA, TSA | 0.003 |
| 3g | Human density | Coral | Structure, MPA,TSA | 0.011 |
| 4 | Human density | TSA | - | 0.579 |
| 4a | Human density | TSA | Structure | 1.296 × 10^−3^ |
| 4b | Human density | TSA | MPA | 1.317 × 10^−5^ |
| 4c | Human density | TSA | Coral | 0.248 |
| 4d | Human density | TSA | Structure, MPA | 2.988 × 10^−5^ |
| 4e | Human density | TSA | Structure, Coral | 4.439 × 10^−4^ |
| 4f | Human density | TSA | MPA, Coral | 3.298 × 10^−6^ |
| 4g | Human density | TSA | Structure, MPA, Coral | 1.299 × 10^−5^ |
| 5 | Structure | MPA | - | 1.332 × 10^−14^ |
| 5a | Structure | MPA | Human density | 6.959 × 10^−13^ |
| 5b | Structure | MPA | Coral | 1.300 × 10^−11^ |
| 5c | Structure | MPA | TSA | 8.572 × 10^−14^ |
| 5d | Structure | MPA | Human density, Coral | 1.464 × 10^−9^ |
| 5e | Structure | MPA | Human density, TSA | 1.206 × 10^−9^ |
| 5f | Structure | MPA | Coral, TSA | 1.662 × 10^−10^ |
| 5g | Structure | MPA | Human density, Coral, TSA | 1.169 × 10^−5^ |
| 6 | Structure | Coral | - | 9.676 × 10^−6^ |
| 6a | Structure | Coral | Human density | 2.808 × 10^−5^ |
| 6b | Structure | Coral | MPA | 5.588 × 10^−3^ |
| 6c | Structure | Coral | TSA | 4.594 × 10^−5^ |
| 6d | Structure | Coral | Human density, MPA | 0.017 |
| 6e | Structure | Coral | Human density, TSA | 3.396 × 10^−5^ |
| 6f | Structure | Coral | MPA, TSA | 0.022 |
| 6g | Structure | Coral | Human density, MPA, TSA | 0.053 |
| 7 | Structure | TSA | - | 0.052 |
| 7a | Structure | TSA | Human density | 2.294 × 10^−4^ |
| 7b | Structure | TSA | MPA | 0.100 |
| 7c | Structure | TSA | Coral | 0.124 |
| 7d | Structure | TSA | Human density, MPA | 0.082 |
| 7e | Structure | TSA | Human density, Coral | 2.356 × 10^−4^ |
| 7f | Structure | TSA | MPA, Coral | 0.226 |
| 7g | Structure | TSA | Human density, MPA, Coral | 0.174 |
| 8 | MPA | Coral | - | 7.831 × 10^−6^ |
| 8a | MPA | Coral | Human density | 2.692 × 10^−6^ |
| 8b | MPA | Coral | Structure | 4.565 × 10^−3^ |
| 8c | MPA | Coral | TSA | 1.414 × 10^−7^ |
| 8d | MPA | Coral | Human density, Structure | 2.164 × 10^−3^ |
| 8e | MPA | Coral | Human density, TSA | 9.846 × 10^−9^ |
| 8f | MPA | Coral | Structure, TSA | 1.277 × 10^−4^ |
| 8g | MPA | Coral | Human density, Structure, TSA | 7.338 × 10^−5^ |
| 9 | MPA | TSA | - | 3.145 × 10^−7^ |
| 9a | MPA | TSA | Human density | 3.206 × 10^−11^ |
| 9b | MPA | TSA | Structure | 1.369 × 10^−6^ |
| 9c | MPA | TSA | Coral | 6.433 × 10^−9^ |
| 9d | MPA | TSA | Human density, Structure | 4.695 × 10^−8^ |
| 9e | MPA | TSA | Human density, Coral | 1.762 × 10^−13^ |
| 9f | MPA | TSA | Structure, Coral | 6.320 × 10^−8^ |
| 9g | MPA | TSA | Human density, Structure, Coral | 3.287 × 10^−9^ |
| 10 | Coral | TSA | - | 0.774 |
| 10a | Coral | TSA | Human density | 0.278 |
| 10b | Coral | TSA | Structure | 0.785 |
| 10c | Coral | TSA | MPA | 2.957 × 10^−3^ |
| 10d | Coral | TSA | Human density, Structure | 0.114 |
| 10e | Coral | TSA | Human density, MPA | 4.964 × 10^−4^ |
| 10f | Coral | TSA | Structure, MPA | 0.013 |
| 10g | Coral | TSA | Human density, Structure, MPA | 3.027 × 10^−3^ |

Table S2: **Conditional independence tests for causal discovery.** We use G^2^ test of conditional independence, where we reject independence when the p-value < 0.05.
